# Male coercive mating in externally fertilizing species: male coercion, female reluctance and explanation for female acceptance

**DOI:** 10.1038/srep24536

**Published:** 2016-04-18

**Authors:** Yukio Matsumoto, Takeshi Takegaki

**Affiliations:** 1 Center for Coastal Fisheries and Aquaculture, Tohoku National Fisheries Research Institute, Fisheries Research Agency, Iwate, Japan; 2 Graduate School of Fisheries and Environmental Sciences, Nagasaki University, Nagasaki, Japan

## Abstract

Male coercive mating exerts a strong evolutionary pressure on mating-related traits of both sexes. However, it is extremely rare in externally fertilizing species probably because the male mating behaviour is incomplete until females release their eggs. Here we showed that males of the externally fertilizing fish *Rhabdoblennius nitidus* coercively confine females to the nests until spawning, and investigated why females accept male coercive mating. The females entered the males’ nests following male courtship displays, but they usually tried to escape when there were no eggs because males tended to cannibalize all the eggs when there were few. Most males that used small, tight nests acquired new eggs but with experimentally enlarged nests, 90% of the males without eggs failed to confine the females. Spawning tended to occur during the early/late spawning period in nests with no eggs (i.e. male coercive mating). In the nests where the first eggs were deposited in the early period, subsequent matings with other females were more likely to occur, whereas in the late period, most parental care of the eggs failed without additional matings. The females that spawned in the late period may have been compelled to accept male coercive mating due to time constraints.

Mate preference is more likely to evolve in females^1^; however, it is also true that females often suffer from coercive mating with unwanted males^2^,^3^. Females tend to resist unwanted mating, but they are finally compelled to accept it in most cases. Therefore, male coercive mating is a significant phenomenon which could have affected the evolution of female mate preference^4^, and it may exert strong evolutionary pressure on the morphological and behavioural traits related to mating, as well as on the mating strategies of both sexes^3^,^5^. Thus, if there is a possibility of coercive mating in focal animals, it is important to determine whether the mating behaviour is truly coercive to understand the evolutionary mechanisms that underlie the mating strategies of species^6^. However, these evaluations are difficult if there is no obvious female resistant behaviour or morphology. For example, male garter snakes elicit hypoxic stress in females simply by laying on their bodies, thereby inducing cloacal opening to allow intromission^6^. Moreover, male behaviours such as biting and holding appear to force females to accept them^3^,^7^,^8^, but they can also be regarded as an essential stimulation required for successful spawning^9^,^10^.

Most examples of male coercive mating have been reported in internally fertilizing species^3^,^11^, and they are extremely rare in externally fertilizing species (e.g. waterfrog^8^ and salmon fish^12^). This is probably because of the fundamental structural differences between these modes of fertilization. In internally fertilizing species, males can forcibly mate with females by transferring their sperm to the female copulatory organ, whereas in externally fertilizing species, males need to force females to release their eggs to achieve coercive mating. One likely reason for the few reports of male coercive mating in externally fertilization species is that coercive mating depends on the occurrence of female spawning. This interpretation raises an intriguing question about why females spawn eggs when mating with non-preferred males. After females spawn their eggs, the opportunity for counter-adaptations following male coercive mating is severely limited (but see ref. 11), whereas in species with internal fertilization, various female counter-adaptations are known, such as forceful termination of copulation, sperm selection and reducing the number of offspring produced (i.e. cryptic female choice)^11^.

From the female perspective, male coercive mating is divided into two types: unilateral male enforcement and female acceptance of mating based on a consideration of the costs associated with the acceptance (e.g. low quality males^13^) or avoidance (e.g. male harassment^14^,^15^ and punishment^16^) of coercive mating. It is important to discriminate between these types to understand the underlying mechanisms of sexual conflict over coercive mating. However, in internally fertilizing species, identifying female acceptance appears to be nearly impossible because there is no distinct boundary in the phenotypes between acceptance and enforcement. In contrast, in externally fertilizing species, the boundary can be discriminated clearly based on the process of female spawning. This specific feature allows us to show that females are being coerced to mate but also that they accept it, albeit reluctantly, as well as examining the reasons why females accept male coercive mating in a quantitative manner.

*Rhabdoblennius nitidus* (Blennidae) is a marine fish with external fertilization, which inhabits rocky intertidal shores. The males occupy small rock holes or vacant gastropod shells as spawning nests, and they accept eggs from several females^17^. The eggs are attached inside the nests and tended by the male alone until hatching (ca. 7 days)^17^. The male reproductive activity varies between courtship and parental phases according to androgen-mediated brood cycling^18^. After the male has acquired eggs from the first female, it exhibits courtship displays for only the next 2 days because of the shift from courtship to the parental phase^18^. If the male acquires few eggs during the courtship phase, all of the eggs are usually eaten by the male, probably due to the expected low reproductive return from the parental care investment^19^. Therefore, females prefer males with more eggs in their nests^20^. Male coercive-like mating often occurs when a female enters an eggless nest following male courtship displays. The nesting male seems to push the female deeper into the nest (Fig. 1a) and to plug the nest by bending its body (Fig. 1b,c). However, it is difficult to demonstrate whether these male behaviours are coercive mating because they appear to be part of the spawning behaviour, such as sperm release behaviour. A simple way to provide evidence of male coercive mating is to disable male traits that are specialized for coercive mating, as demonstrated in some insects^21^,^22^ but such specific morphological traits are not observed in *R. nitidus* males. In some substrate brooding fishes including *R. nitidus*, nesting males show the strong preference for a size-matched tight nest^23^,^24^,^25^ probably due to the advantage for guarding against egg predators^23^. We focused on the male size-assortative nest preference and hypothesized that a narrow gap between the male’s body and the nest’s inner wall may also be beneficial to the male if the gap allows it to confine females in the nest. Therefore, the first aim of this study was to experimentally demonstrate that *R. nitidus* males coercively confine reluctant females in the nests until spawning has occurred.

When females accept male coercive mating in *R. nitidus*, this means that they spawn in eggless nests. Therefore, as mentioned above, the primary cost of female acceptance in male coercive mating is a high rate of failure for male parental care due to the whole clutch filial-cannibalism caused by the small number of eggs in the nests^19^. The forced females may be able to avoid the failure of male egg care if the number of eggs in the nest is increased through additional spawning by other females. The females of this species have a strong preference for males that are mating with the other females (i.e. mate-choice copying), so additional spawning is expected to be triggered by coercive mating itself. In this study, we assumed that the possibility of additional mating would be affected by the remaining spawning time period, which is strictly limited to within the same day in this species, and we investigated the frequency of female acceptance of male coercive mating during the spawning time period. Moreover, as predicted by the mathematical model of mate-sampling^26^, ^27^, females are expected to accept mating with non-preferred males when the available spawning time period is limited. If this is applicable to *R. nitidus* females, then they may readily accept coercive mating because the remaining spawning time is reduced. To test these hypotheses, we analysed female mate-sampling behaviour surveyed in the wild.

## Results

### Experimental demonstration of male coercive mating

The experiments were performed for 5 and 6 males using small nests with and without eggs, respectively, and for 9 and 10 males using large nests with and without eggs, respectively. During the course of the experiments, female visits and intrusions to the nests were observed 12 and 11 times for the small nests with and without eggs, respectively, and 20 and 21 times for the large nests with and without eggs, respectively. Most of the females that had intruded into the nests immediately (within 2 sec) attempted to escape from the nests (55/64 intrusions), but 11 of them were confined by the males and then spawned in the nests. The female escape attempts occurred significantly less frequently in the nests with eggs (small nests, 8/12 visits; large nests, 16/20 visits) compared with the nests without eggs (small nests, 10/11 visits; large nests, 21/21 visits), irrespective of nest and male size (likelihood-ratio test, the presence of eggs, *χ*^*2*^ = 11.32, *p* < 0.001; nest size, *χ*^*2*^ = 0.21, *p* > 0.05; male size, *χ*^*2*^ = 0.68, *p* > 0.05). In the case of the nests with eggs, the number of eggs adjusted by egg removal manipulation (details in Methods) did not affect on the occurrence of female escape attempt from the nests (likelihood-ratio test, *χ*^*2*^ = 1.38, *p* > 0.05). The females that attempted to escape from the nests were pushed back by the mouths by the nesting males (73% of 55 attempts), whereas this male threat behaviour was not observed when females stayed in the nests (all 9 cases; Fisher’s exact test, *p* < 0.0001).

**Figure 1:**
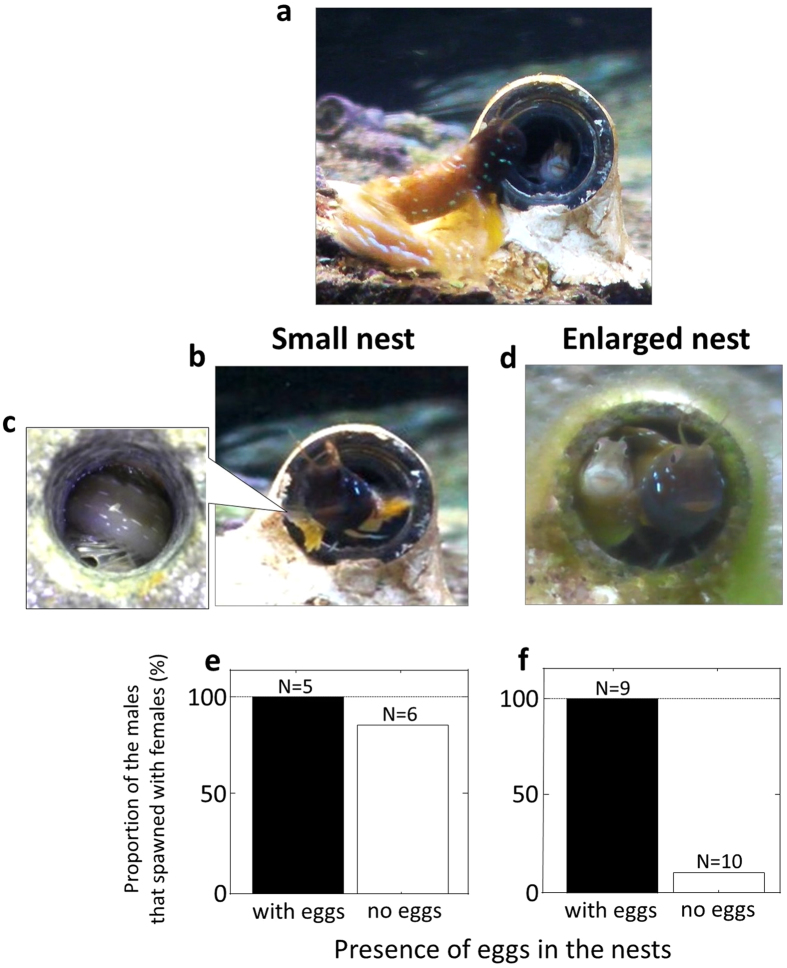
(**a**) Nesting male (left) pushing a female back into the nest (small double-layered nest) from the outside. (**b**,**c**) Nesting male plugging the nest by bending its body in the nest. Image (**c**) shows a view from behind the nest where the bottom plug was removed temporarily for the photograph. (**d**) Female passing through the gap between the male’s body and the inside wall of the enlarged nest. Comparison between the proportion of males that spawned with females when they had eggs and when they did not have eggs in the small nest (**e**) and enlarged nest (**f**).

The proportion of the males that spawned with females was different among four experimental conditions (Fisher’s exact test, *p* < 0.0005; Fig. 1e,f), but was not affected by male body size (GLM with likelihood ratio test, *df* = 1, *χ*^*2*^ = 2.19, *p* > 0.05). Most of the males that used small nests acquired new eggs regardless of whether eggs were present in the nests initially (Tukey’s WSD test, *p* > 0.05; Fig. 1e). All of the males with eggs in the enlarged nests acquired new eggs, whereas 90% of the males without eggs failed to confine females (Fig. 1d), and thus they did not acquire new eggs (*p* < 0.05; Fig. 1f). The proportion of males that mated successfully did not differ between males that used large nests with eggs and males that used small nests with no eggs (*p* > 0.05; Fig. 1e,f). The males that used large nests with no eggs had significantly less mating success than the males that used small nests with eggs (*p* < 0.05) and with no eggs (*p* < 0.05; Fig. 1e,f).

### Explanation for female acceptance of male coercive mating

**Figure 2:**
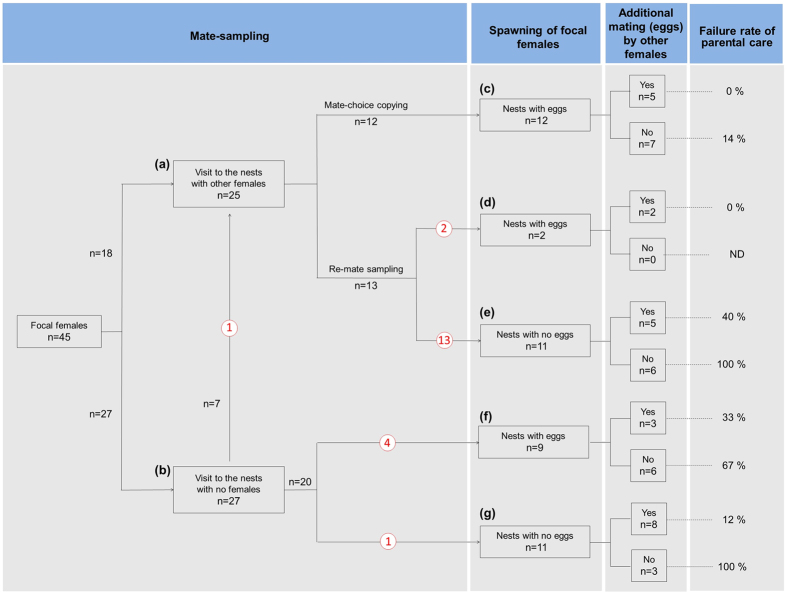
Flowchart illustrating the mate-sampling behaviour of the focal females and the fates of their eggs. For female mate-choice copying, the mate-sampling behaviour was initially divided into two types based on the presence of other females in the nests that the focal females visited (**a**,**b**). The encircled numbers indicate the number of visits to the eggless nests without spawning. The following chart shows the presence or absence of eggs in the nests where females spawned (**c**–**g**), the presence or absence of additional mating (eggs) by other females in the nests, and the failure rate of parental care for their eggs.

In this study, we observed 45 female mate-samplings that ended with spawning (Fig. 2). Twenty-five of these females visited nests where other females were already present (i.e. attempts at mate-choice copying; Fig. 2a), among which 12 spawned in these nests (Fig. 2c). However, the remaining 13 females left without spawning and resumed mate-sampling, where only 2 spawned in the nests with eggs (Fig. 2d), but 11 spawned in the nests with no eggs (Fig. 2e). Among the other 20 females that did not visit the nests with other females (Fig. 2b), 9 females spawned in the nests with eggs (Fig. 2f) and 11 in the nests with no eggs (Fig. 2g).

**Figure 3:**
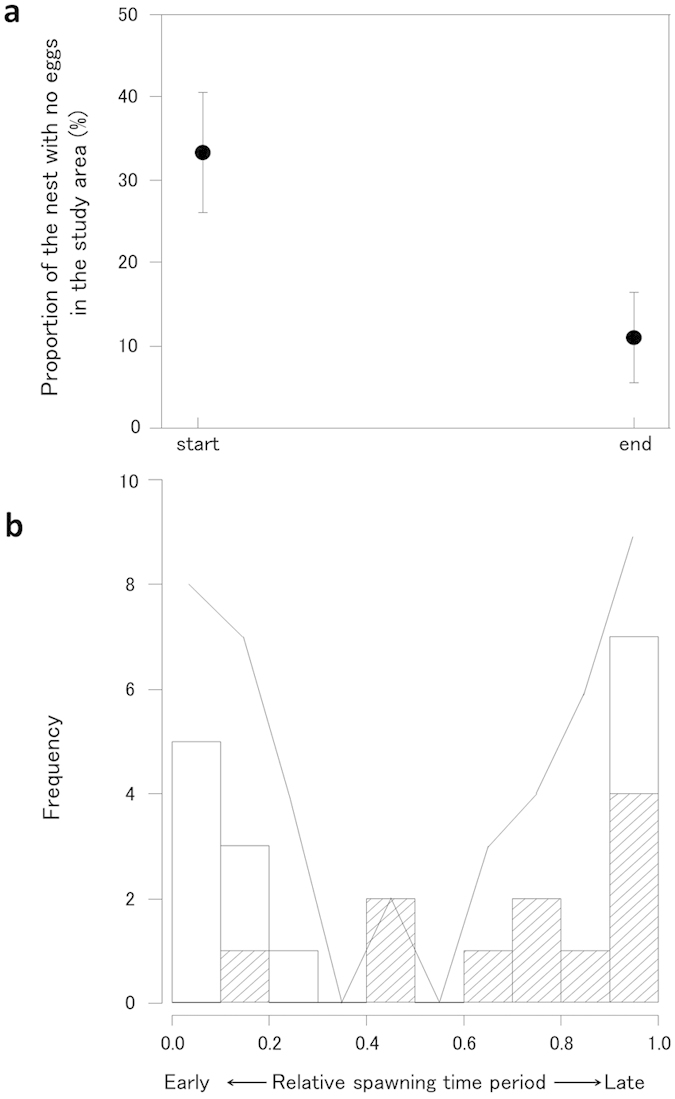
(**a**) Mean (±95% confidence level) proportion of nests with no eggs in the study area at the start and end of the spawning time period (n = 30). (**b**) The frequency of female visits to nests with no eggs (solid line), and the frequency of spawning in the nests with no eggs by females that had initially visited another nest with other females (shaded bar, n = 11; also see Fig. 2e) and by females that had visited the same nests with no females (open bar, n = 11; also see Fig. 2g) during the spawning time period.

**Figure 4:**
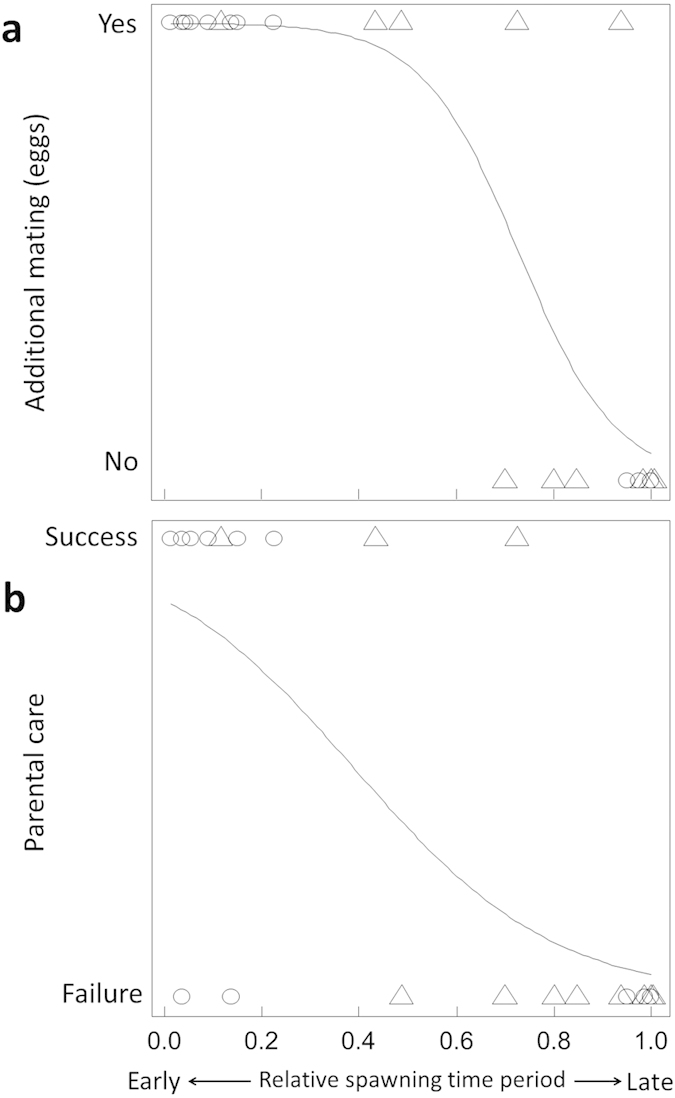
Relationships between the relative spawning time period and (**a**) the occurrence of additional mating by other females and (**b**) the failure rate of parental care in eggless nests where the focal female spawned. Open circles and triangles indicate the females that visited nests with no females and nests with other females, respectively.

The frequencies of female visits and subsequent spawning in eggless nests were relatively high during the early and late spawning periods (Fig. 3b), although eggless nests were present throughout the spawning time period (Fig. 3a). In the cases of spawning in the eggless nests, the occurrence of additional mating by other females decreased (likelihood-ratio test, *χ*^*2*^ = 19.80, *p* < 0.001; Fig. 4a) and failure rate of parental care increased (*χ*^*2*^ = 12.03, *p* < 0.0001; Fig. 4b) as the remaining spawning period in the day decreased. The additional mating (eggs) following spawning in eggless nests decreased the failure rate of parental care (likelihood-ratio test, *χ*^*2*^ = 13.72, *p* < 0.0001; Fig. 2). Even without additional mating, spawning in nests that already contained eggs resulted in a relatively low failure rate of parental care (38%: 5/13), which was equivalent to that in the eggless nests with additional mating (23%: 3/13; Fisher’s exact test, *p* > 0.05).

## Discussion

In this study, we showed that male coercive mating occurs in species with external fertilization based on experimental demonstrations of the sexual conflict over mating. Females of *R. nitidus* often attempted to escape from the visited nests without spawning, and nesting males attempted to confine those females in the nests and then spawned with them. The female escape attempts were significantly more frequent in eggless nests, and when eggs were present, females spawned even in the enlarged nests, from which it is easy to escape. Since failure of male parental care in this species is likely to occur when egg number is small^19^, the female escape from eggless nests may be adaptive. On the other hand, males succeeded in forcing spawning in the eggless nests by preventing females’ escape only if they used small tight nests. The results of our experiments well demonstrated the female reluctance and male coerciveness over mating. The occurrence of female escape attempt even in the presence of eggs may suggest the presence of other factors affecting female mate or nest choices, such as male body size, nest size and egg number, which may be associated with parental care success, but these possibilities were rejected in this study. Although the reason for the female escape attempts from the nests with eggs is unclear, the fact that in the enlarged nests females resulted in spawning only when eggs were present implies that the presence of eggs affected whether females spawned eggs in the nests.

Male coercive mating exerts a strong evolutionary pressure on male morphological traits and mating-related behavioural traits, as well as female traits for resistance and counter-adaptation^3^,^11^, such as male grasping and female anti-grasping structures in insects^28^,^29^. In *R. nitidus*, the male preference for size-assortative nests may be an example of this type of trait^23^. The use of size-assortative nests appears to be effective in egg guarding against predators^23^, but it may be disadvantageous in egg accommodation compared with the use of size mismatched large nests, as shown in some substrate-breeding fishes^30^,^31^. However, for males, the first mate acquisition by coercive mating is an almost essential process for obtaining successive additional matings via female mate-choice copying^20^, and thus it is a critically important process in determining their reproductive success. The high effectiveness of size-assortative nests for confining females, in the present study, strongly suggests that the male preference for using size-assortative nests may have evolved as a trait for coercive mating. In addition to the male trait, a female mate-choice copying behaviour^20^ appears to be associated with male coercive mating. This is a certain tactic for choosing nests with eggs but without entering the nests to confirm the presence of eggs, thereby avoiding the risk of confinement for females. It is unclear whether the female mate-choice copying has evolved in this species as a counter-adaptation against male coercive mating, but most females could avoid the failure of male egg care by mate-choice copying (Fig. 2).

A highly puzzling question related to male coercive mating in externally fertilizing species is, why females spawn. Coercive mating by *R. nitidus* males could not occur if females simply did not lay their eggs even if they are confined to the nests. Thus, female mating acceptance may be affected by the costs of *avoiding* and *accepting* male coercive mating. One evident and serious cost of avoidance is male harassment. If females manage to leave the nests without laying eggs, they are usually chased and bitten by the nesting male and some are seriously injured^20^. The male’s attacks may also have the effect of making females hesitant about leaving the nests. In addition, a significant cost of female acceptance of coercive mating is a high risk of failure in the male parental care of eggs. Males of this species usually cannibalize all of the eggs when there are few eggs in the nests^19^, so female spawning in eggless nests has a high risk of egg care failure unless additional matings occur, as shown in the present study (Fig. 2). Interestingly, female spawning in the eggless nests (i.e. male coercive mating) occurred more frequently during the early and late spawning time periods. First, the eggs spawned during the early time period were tended with a high success rate (Fig. 4b) because additional eggs laid by other females were more likely to be added (Fig. 4a), which may be attributable to the length of the remaining spawning time period available on the same day. Therefore, females may readily accept male coercive mating during the early time period due to the low risk of parental care failure (i.e. low cost of acceptance). Second, the frequent spawning in eggless nests during the late time period may have been attributable to rushed spawning immediately before the end of the spawning time period. During the spawning time period, females usually tried to copy the mate choice of others, and thus several females were often observed waiting for the ongoing spawning to end around a single nest. Each female required from several dozen minutes to over one hour for egg deposition, so the waiting females became impatient and began to visit nearby nests with no eggs. Thus, late spawning in the eggless nests was attributable mainly to these wandering females (shaded bar in Fig. 3b). As predicted by the mathematical models^26^,^27^, if the available mate-sampling and spawning times are limited, females may accept unwanted mating despite substantial costs. However, it was intriguing that the parental care of eggs spawned during the late period resulted in a high failure rate, which was probably due to the absence of additional eggs (Fig. 4). Why did the females spawn eggs during the late time period despite the high risk of parental care failure? One possible explanation is that females may have needed to spawn their eggs on the same day because delayed spawning might cause a decrease in the egg fertilization rate as the eggs could become overripe, as shown in other fishes^32^,^33^.

Male coercive mating is a highly significant evolutionary phenomenon, but it has received very little attention in externally fertilizing species because female egg release is required to achieve coercive mating. This is the first study to provide direct empirical evidence that male coercive mating occurs in externally fertilizing species. Moreover, based on the female egg release process, we showed that females yielded to force but they also made explicit decisions to accept coercive mating, which depended on the specific situation. These fertilization mode-specific characteristics will facilitate a new approach to understand the evolution of male coercive mating, as well as further studies in internally fertilizing species.

## Methods

### Experimental demonstration of male coercive mating

The field experiments were conducted from August to September 2011 in four intertidal pools (at low tide: 2.9–12 m^2^ in area, 20–50 cm) on the Mie coast, Nagasaki Prefecture, Kyushu, Japan (32° 45′ N, 129° 47′ E). In this study, we observed 30 nesting males in total, which were captured using a hand net. The collected fishes were anaesthetised by immersion in 600 ppm MS-222, and their standard length (SL) was measured (mean SL ± SD = 56.07 ± 6.60 mm, range = 45.60–67.20 mm). They were identified by the differences in colour and position of the paint injected hypodermically on each side of body. The marked fishes were allowed to recover from anaesthesia in aerated seawater, before they were returned to their own nests. All males survived this procedure. *Rhabdoblennius nitidus* males preferentially use highly size-assortative nests for spawning site^23^, so nest size manipulation experiments were used to control the degree of difficulty when nest-holding males confined females. To make a male use a size-mismatched large nest, acrylic double-layer pipes were used as an artificial nest (Fig. S1), where a small pipe nest (internal diameter: 11 mm, external diameter: 17 mm, length: 65 mm) was inserted within a large one (17 mm, 22 mm, 65 mm). In our previous studies at this study site, we found that males preferred to use small single layer pipe nests (internal diameter: 11 mm) and they rarely used the medium ones (14 mm)^19^. The small pipe nests were physically available for females of all body sizes. The artificial nests were fixed onto the sea floor using an epoxy resin. An attachable translucent plastic sheet was set on the inner surface of the nest side wall to allow us to monitor the number of eggs deposited in the nest by withdrawing the sheet^19^. If males occupied the artificial nests before the spawning time period on a given day, the small pipe was removed from the large pipe to increase the nest size (Fig. S1). To control for the effect of the nest manipulation on male behaviour, the small pipe was withdrawn and reinserted before the observations of the males using small pipe nests without changing nest size.

We tested the following four treatments in this study: males using small nests with eggs and without eggs, and males using large nests with eggs and without eggs. In *R. nitidus*, male mating success is affected by the male courtship intensity, which begins to decrease 2 days after the first spawning^18^. Therefore, males tending 1-day-old eggs were used as the ‘males with eggs’ in these experiments. In addition, females prefer to spawn in nests with vacant spawning space at the centre and deep sites rather than at the entrance site^34^. To make a preferable spawning space in the nests, the positions and numbers of egg in the nest were adjusted (mean number of remaining eggs ± SD = 810 ± 90 eggs, range = 600–901 eggs) by removing eggs on the egg-sheet. The egg removal manipulations may not affect male mating success because there is no significant difference in male courtship intensity between males with and without eggs^18^. After the nest manipulation, the reproductive behaviour of the focal nesting males were recorded using an underwater digital video camera (Xacti DMX-WH1; Sanyo, Osaka, Japan) during the spawning time period each day. In general, the females did not emerge from the nests until the end of spawning, so females putting their heads outside of the nests were treated as females attempting to escape from the nests in this study. Male behaviour pushing females with their mouth was recorded as a male threat behaviour. After the females left the nests, the egg sheet was withdrawn to confirm whether the females had spawned eggs in the nests.

A generalized linear model (GLM) with binomial distribution was used to analyze the effects of the presence of eggs, nest size, and male body size (SL) on the occurrence of female escape attempt. The statistical significance of the explanatory variables were tested using likelihood-ratio test. The effect of female escape attempt on the occurrence of male threat behaviour was analysed using Fisher’s exact test. The effect of male body size (SL) on the male mating success was analysed using combination of GLM with binomial and likelihood-ratio test. The proportion of the males that mated successfully with females was compared using Fisher’s exact test. In addition, Tukey’s WSD tests were used to determine the differences among groups. The statistical tests were conducted using R-2.15.3 (http://www.r-project.org/).

### Explanation for female acceptance of male coercive mating

To explain female acceptance of male coercive mating, we investigated female mate-sampling behaviours by snorkelling in intertidal pools on the Mie coast from July to September 2011. Spawning usually occurred during the early morning flood and ebb tides, which lasted approximately 2 hours each day, depending on the tidal cycle (average time period ± SD = 136 ± 72 min; range = 30–286 min, n = 30 days). When the spawning time arrived, females began to move from the subtidal zone into the intertidal study pools where 48 small pipe nests (see above) were occupied by males, and they then selected nest-holding males. In this study, the spawning time period was defined as the time when the first female visited the study pool until the time when the last female entered the spawning nest. Before the spawning time period on a given day, we examined the presence of eggs in all of the artificial nests occupied by males by withdrawing the plastic sheets from the nests. We then observed focal females during the spawning time period and recorded the nests where they visited, entered and spawned, as well as the times when these events occurred, and the presence of other females in the nests. The presence of additional eggs spawned by the focal females was examined by withdrawing the egg-sheet after they left the nests. To investigate the additional eggs spawned by other females and the proportion of the nests with no eggs in the study pools, all of the nest sheets were again checked after the spawning time period. To examine the effects of male coercive mating on female reproductive success, survival of the eggs spawned by each focal female was investigated. Since it is nearly impossible to distinguish one clutch from the other clutches spawned in the same nest, we used occurrence rate of the loss of all eggs in the nests (mostly caused by the total filial-cannibalism by male parent) to evaluate survival rate of the focal female’s eggs.

In this study, 45 females that had spawned up to the end of the spawning time period were used in the analysis. To examine the timings of female visits and spawning in the eggless nests (i.e. timing of acceptance of male coercive mating) during the spawning time period, we employed the elapsed time relative to the total length of the spawning time period. Similarly, to examine the effects of the timing of female acceptance of male coercive mating on the probability of additional mating by other females following coercive mating and the fate of eggs spawned via coercive mating, we analysed the relationships between the relative elapsed time and the presence of additional eggs by other females, and the parental care success using a generalized linear model (GLM) with a binomial distribution. The performance of the GLM was measured using likelihood-ratio test statistics. We compared the failure rate of parental care (i.e., the number of the males that lost all eggs before hatching/total number of tested males) with spawning in the nests that already contained eggs and spawning in the eggless nests with additional mating using Fisher’s exact test. The analyses were conducted using R-2.15.3 (http: //www.r-project.org/).

### Ethical statement

This research was performed in accordance with the guideline for ethological studies by the Japan Ethological Society (www.ethology.jp/guideline.pdf) and the guidelines for the use of fishes in research by the Ichthyological Society of Japan (http://www.fish-isj.jp/english/guidelines.html). No permits were needed from the Japanese government for experiments involving *R. nitidus*.

## Additional Information

**How to cite this article**: Matsumoto, Y. and Takegaki, T. Male coercive mating in externally fertilizing species: male coercion, female reluctance and explanation for female acceptance. *Sci. Rep.* **6**, 24536; doi: 10.1038/srep24536 (2016).

## Supplementary Material

Supplementary Information
