# Supplementary material for: Male coercive mating in externally fertilizing species: male coercion, female reluctance and explanation for female acceptance
Source: Sci Rep. 2016 Apr 18;6:24536. doi: 10.1038/srep24536 (PMC4834473; doi:10.1038/srep24536)

Male coercive mating in externally fertilizing species: male coercion, female reluctance and explanation for female acceptance

Matsumoto, Y. & Takegaki, T.

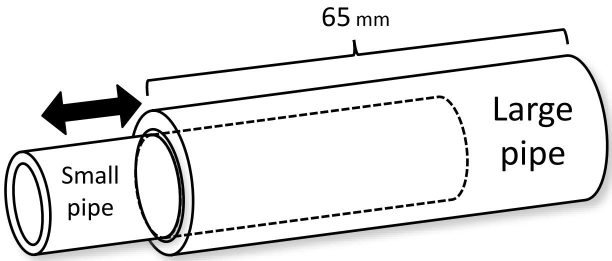

Supplement: Supplementary Information [file srep24536-s1.pdf]
